# Identification of Keratin 23 as a Hepatitis C Virus-Induced Host Factor in the Human Liver

**DOI:** 10.3390/cells8060610

**Published:** 2019-06-18

**Authors:** Volker Kinast, Stefan L. Leber, Richard J. P. Brown, Gabrielle Vieyres, Patrick Behrendt, Constanze Eßbach, Pavel Strnad, Florian W. R. Vondran, Markus Cornberg, Cora Wex, Thomas Pietschmann, Johannes Haybaeck, Daniel Todt, Eike Steinmann

**Affiliations:** 1 Ruhr University Bochum, Faculty of Medicine, Department for Molecular and Medical Virology, 44801 Bochum, Germany; volker.kinast@rub.de; 2 Institute of Experimental Virology, Twincore Centre for Experimental and Clinical Infection Research, a joint venture between the Medical School Hannover (MHH) and the Helmholtz Centre for Infection Research (HZI), 30625 Hannover, Germany; gabrielle.vieyres@twincore.de (G.V.); patrick.behrendt@twincore.de (P.B.); thomas.pietschmann@twincore.de (T.P.); 3 Department of Pathology, Otto-von-Guericke University of Magdeburg, 39106 Magdeburg, Germany; stefan.leber@medunigraz.at (S.L.L.); constanze.essbach@med.ovgu.de (C.E.); johannes.haybaeck@med.ovgu.de (J.H.); 4 Diagnostic & Research Center for Molecular BioMedicine, Institute of Pathology, Medical University of Graz, 8036 Graz, Austria; 5 Department of General, Visceral, Vascular and Transplantation Surgery, Otto-von-Guericke University of Magdeburg, 39106 Magdeburg, Germany; cora.wex@med.ovgu.de; 6 Division of Veterinary Medicine, Paul Ehrlich Institute, 63225 Langen, Germany; Richard.Brown@pei.de; 7 Department of Gastroenterology, Hepatology and Endocrinology, Hannover Medical School, 30625 Hannover, Germany; Cornberg.Markus@mh-hannover.de; 8 German Center for Infection Research (DZIF), Partner Site Hannover-Braunschweig, 30625 Hannover, Germany; Vondran.Florian@mh-hannover.de; 9 Department of Internal Medicine III, University Hospital Aachen, 52074 Aachen, Germany; pstrnad@ukaachen.de; 10 ReMediES, Department of General, Visceral and Transplantation Surgery, Hannover Medical School, 30625 Hannover, Germany; 11 Department of Pathology, Neuropathology, and Molecular Pathology, Medical University of Innsbruck, 6020 Innsbruck, Austria

**Keywords:** hepatitis C virus (HCV), keratin 23, host factor, virus–host interaction

## Abstract

Keratin proteins form intermediate filaments, which provide structural support for many tissues. Multiple keratin family members are reported to be associated with the progression of liver disease of multiple etiologies. For example, keratin 23 (KRT23) was reported as a stress-inducible protein, whose expression levels correlate with the severity of liver disease. Hepatitis C virus (HCV) is a human pathogen that causes chronic liver diseases including fibrosis, cirrhosis, and hepatocellular carcinoma. However, a link between KRT23 and hepatitis C virus (HCV) infection has not been reported previously. In this study, we investigated KRT23 mRNA levels in datasets from liver biopsies of chronic hepatitis C (CHC) patients and in primary human hepatocytes experimentally infected with HCV, in addition to hepatoma cells. Interestingly, in each of these specimens, we observed an HCV-dependent increase of mRNA levels. Importantly, the KRT23 protein levels in patient plasma decreased upon viral clearance. Ectopic expression of KRT23 enhanced HCV infection; however, CRIPSPR/Cas9-mediated knockout did not show altered replication efficiency. Taken together, our study identifies KRT23 as a novel, virus-induced host-factor for hepatitis C virus.

## 1. Introduction

Keratins (KRTs) are predominantly known for their characteristic of forming intermediate filaments within epithelial cells, providing tissues with mechanical support. Indeed, several KRT-linked diseases lead to tissue fragility [1,2]. However, KRTs are not exclusively associated with epithelia- and tissue-related diseases. Genetic mouse models highlighted the function of KRTs in protecting hepatocytes from apoptosis and necrosis, and mutations within the KRT8 and KRT18 genes are linked to the progression of liver disease of multiple etiologies [3]. The expression of a variety of KRTs is triggered by inflammatory cytokines, indicating an important role for these proteins during the cellular stress response [4]. In particular, KRT18 release into the extracellular space is mediated by hepatocellular damage and is therefore a commonly-used, non-invasive marker of liver diseases [5]. Additionally, KRT23, strongly upregulated in several human cancers, was suggested to be a ductular reaction marker, since its levels correlate with liver disease severity [6,7,8]. Furthermore, viral infections have been associated with the modulation of KRTs. In particular, the progression of chronic hepatitis B virus infection is considered to associate with the phosphorylation of KRT18 [9], whereas the cleavage of KRT18 was shown to correlate with the stress response in livers of chronic hepatitis C patients [10].

Hepatitis C virus (HCV) is an enveloped positive strand RNA virus belonging to the family of *Flaviviridae*. HCV strains are classified into seven genotypes (GT1–7) differing up to 30% at the nucleotide level [11]. Its 9.6-kb genome consists of one open reading frame, flanked by a 5′ and a 3′ untranslated region. An internal ribosomal entry site enables the expression of the polyprotein, which is subsequently processed into seven non-structural and three structural proteins [12]. Infection with HCV causes acute hepatitis and progresses to a chronic infection in most cases. Chronic hepatitis C (CHC) patients represent a patient population at high risk for development of serious liver diseases, including steatosis, cirrhosis, and hepatocellular carcinoma [13]. Although direct-acting antivirals were introduced in 2014 and have replaced PEGylated interferon-α (pegIFN-α) and ribavirin (RBV) as the first choice of anti-HCV treatment, this hepatotropic pathogen is still a global health burden with 71 million infected people, especially since the majority of HCV-infected patients are unaware of their infection status [13].

In this study, we analyzed the expression of different KRTs, including KRT23, in vivo and ex vivo upon HCV infection and pegIFN-α therapy. In addition, we analyzed the KRT23 levels in direct acting antiviral (DAA)-treated HCV-infected patients before and after viral clearance. Furthermore, we generated KRT23 expressing and KRT23 knockout cells to determine the influence of KRT23 on the HCV life cycle. Taken together, our data indicate that KRT23 is an HCV host factor, whose expression and secretion correlates with the abundance and clearance of the viral infection. 

## 2. Materials and Methods

### 2.1. Cell Culture

The Huh-7.5 cell line and HEK 293T cells were cultured in Dulbecco’s Modified Eagle Medium (1 g/L glucose, Gibco, Thermo Fisher Scientific, Waltham, MA, USA) containing 10% fetal calf serum (FCS, capricorn scientific, Ebsdorfergrund, Germany), 2 mM l-glutamine (Gibco, Thermo Fisher Scientific, Waltham, MA, USA), 0.1 mM of each non-essential amino acid (Invitrogen), 10 U/mL penicillin (Gibco, Thermo Fisher Scientific, Waltham, MA, USA), and 10 μg/mL streptomycin (Gibco, Thermo Fisher Scientific, Waltham, MA, USA) at 37 °C. In addition, 5 μg/mL blasticidin S (InvivoGen, San Diego, CA, USA) or 2.5 µg/mL puromycin (Sigma-Aldrich, St. Louis, MO, USA) were added to cells carrying an integrated lentiviral vector for ectopic expression or CRISPR KO, encoding a blasticidin resistance gene or a puromycin resistance gene, respectively. Primary human hepatocytes (PHHs) were isolated from liver specimens, plated at a density of 1.3 × 10^6^ on collagen-coated 6-well dishes, and kept in hepatocyte culture medium (Lonza, Basel, Switzerland) as described [14].

### 2.2. Compounds and Reagents

2-C-methyladenosine (2-CMA) was kindly provided by Timothy Tellinghuisen (The Scripps, Jupiter, FL, USA). Human IFN-α was purchased from SP Europe/Essex Pharma (IntronA) or from Sigma-Aldrich, St. Louis, MO, USA). His-tagged IFN-λ3 and -λ4 were kindly provided by Rune Hartmann (Aarhus, Denmark) and were used as described previously [15].

### 2.3. Plasmids

pFK-Jc1, pFK_i389-LucEI/NS3-3′-JFH1_dg, pFK-i341PI-Luc/NS3-3/Con1/ET (replicon with E1202T, I1280T, and K1846T mutations), and bicistronic *Renilla* luciferase reporter (RLuc) chimeric HCVcc genomes designated J6/2a/R2a have been described previously [16,17,18,19,20]. For generating CRISPR/Cas9 knock out cell lines, the lentiviral plasmid pLenti CRISPR v2 ccdB was used as described previously [21]. pWPI-empty-BLR and pWPI-3xFLAG-KRT23-BLR (NM_015515.4) were generated by molecular cloning using synthesized gene fragments (gBlocks, IDT). 

### 2.4. Production of Viruses and Pseudoparticles

For production of cell-culture-derived HCV (HCVcc), in vitro transcribed RNA of HCV full-length Jc1 WT and JCR2a were transfected in Huh-7.5 cells. Supernatants, containing HCVcc, were harvested at 48 and 72 h post-electroporation and filtered through a 0.45-μm pore size membrane. Afterwards, HCVcc were concentrated using 100-kDa cutoff Amicon Ultra centrifugal filters (Merck, Darmstadt, Germany).

For production of lentiviral pseudo-particles, HEK 293T cells were transfected with pcz-VSV-G, pCMV-dR8.74, and the respective lentiviral plasmid, by using the PEI method (Carl Roth, Karlsruhe, Germany) or Lipofectamine 2000 (Thermo Fisher Scientific, Waltham, MA, USA). Lentiviral pseudoparticles were harvested 48 and 72 h post-transfection and used for transduction of target cells.

### 2.5. Western Blotting

For Western blot analysis, cells were lysed in RIPA buffer and heated at 95 °C for 5 min with SDS sample buffer. Afterwards, proteins were resolved by SDS-PAGE and transferred to polyvinylidene difluoride membranes by semi-dry electroblotting. Five percent milk in PBS containing 0.05% Tween (PBS-T) was used to block the membranes. Subsequently, membranes were probed with primary antibodies α-FLAG (1:1000, Sigma-Aldrich, Catalogue Number F3165), α-KRT23 (1:2000, Thermo Fisher, Catalogue Number PA5-50198), α-KRT23 (1:1000, Abcam, Catalogue Number ab156569) α-HCV-NS3 #337 mAb (1:1000), α-GAPDH (1:1000, Sigma-Aldrich, Catalogue Number G9545), and α-β-actin (1:20000, Sigma-Aldrich, Catalogue Number A3854) over night at 4 °C, followed by incubation with secondary horseradish peroxidase conjugated antibodies (Sigma-Aldrich) for 1 h at room temperature. For analysis, membranes were incubated with the ECL Plus detection system (GE Healthcare), SuperSignal Femto Substrate (Thermo Fisher), and Pierce™ ECL Plus Western Blotting Substrate (Thermo Fisher). 

### 2.6. Dot Blot

For dot blot analysis, 3 µL of patient plasma were spotted on polyvinylidene difluoride membranes and air-dried for 1 h at room temperature. Membranes were blocked with 5% milk in PBS containing 0.05% Tween (PBS-T) for 1 h at room temperature and subsequently probed with primary antibodies (α-KRT23, 1:1000, kindly provided by Pavel Strnad [8]) over night at 4 °C, followed by incubation with secondary horseradish peroxidase conjugated antibodies (Sigma-Aldrich) for 1 h at room temperature. For analysis, membranes were incubated with the ECL Plus detection system (GE Healthcare) and SuperSignal Femto Substrate (Thermo Fisher). Fiji was used to calculate signal intensities of KRT23 on the different dot blots. Therefore, regions of interest with the same size were selected in all samples, and mean grey values were quantified.

### 2.7. Immunofluorescence Analysis

For immunofluorescence analysis, cells were cultured on cover slips in 24-well plates. After fixation with 3% paraformaldehyde for 10 min, cells were permeabilized by incubation with 0.5% Triton X-100 for 5 min. Subsequently, samples were blocked with 5% FCS in PBS for 1 h at room temperature. For detection of KRT23, samples were incubated with primary antibodies α-FLAG M2 mAb (1:1000, Sigma-Aldrich) over night at 4 °C. Primary antibodies were detected using secondary antibodies conjugated to Alexa Fluor 488 (1:1000, Sigma-Aldrich) by incubation for 1 h at room temperature. Nuclear DNA was stained using DAPI (dilution of 1:10,000). 

### 2.8. Real-Time Quantitative PCR 

To extract total RNA from cell cultures, the Nucleospin RNA II kit (Machery Nagel) was used, according to the manufacturer’s instructions. For synthesis of cDNA, the PrimeScript First Strand cDNA synthesis kit (TaKaRa) was used, following the manufacturer’s instructions. Quantitative PCR was performed with SYBR Premix Ex Taq (Takara), the LightCycler480 system (Roche), and the respective primers. The primer sequences for amplification of the HCV genome and of each gene product (KRT23 and GAPDH) have been described previously [22,23,24].

### 2.9. In Vitro Transcription and Electroporation of Huh-7.5 Cells

*In vitro* transcripts were prepared and used for electroporation-based transfection as described recently [25]. Subsequently, cells were seeded in 96-well plates and subjected to further analysis. 

### 2.10. HCV Infection Assays

The day before inoculation, Huh-7.5 cells and derivatives were seeded at a density of 1 × 10^4^/well in 96-well plates. Cells were inoculated with the respective virus particles at 37 °C for 4 h. Afterwards, the viral inoculum was replaced by fresh culture medium.

### 2.11. Luciferase Assays

To quantify the HCV *Renilla*-reporter virus, cells were washed once with PBS and lysed in H_2_O. After storage at −80 °C for at least 15 min, cell lysates were subjected to luciferase activity measurement. Therefore, samples were incubated with luciferase substrate (1 μmol/L of coelenterazine in PBS, PJK GmbH, Kleinblittersdorf, Germany), and *Renilla* luciferase activity was measured in a luminometer (Lumat LB9507, Berthold). For quantification of the replication activity of firefly reporter subgenomic constructs, cells were washed once with PBS and lysed with lysis buffer (containing 0.1% Triton-X100, 25 mmol/L glycylglycine, 15 mmol/L MgSO_4_, 4 mmol/L EGTA tetrasodium, and 1 mmol/L dithiothreitol, pH 7.8). After storage at −20 °C for at least 30 min, cell lysates were incubated with luciferase substrate (200 µmol/L luciferin, 25 mmol/L glycylglycine, pH 8), and luciferase activity was measured with a luminometer (Lumat LB9507, Berthold).

### 2.12. Generation of CRISPR/Cas9 Knockout Cells

CRISPR/Cas9 knockout cells were generated as described previously [26]. The web tool CHOPCHOP was used to identify two single-guide RNA (sgRNA) sequences [27]. The two sgRNAs, as well as a non-targeting control were cloned into pLenti CRISPR v2 ccdB. For the production of lentiviral pseudo-particles, HEK 293T cells were transfected with pcz-VSV-G, pCMV-dR8.74, and the respective lentiviral plasmid, by using the Lipofectamine 2000 (Thermo Fisher). Lentiviral pseudoparticles were harvested 24 and 48 h post-transfection and used for transduction of target cells. Target cells were inoculated with lentiviruses for 4 h and selected with Puromycin 48 h post-infection. For validation, the sgRNA target sequence in the genomic DNA was analyzed by Sanger sequencing. sgRNAs and corresponding primers for amplification of the genomic DNA are listed in Appendix Table A1.

### 2.13. Statistical Methods

For data analysis, GraphPad Prism 8 software was used. Two-tailed Student’s *t*-test and one-way analysis of variance (ANOVA) adjusted with Dunnett’s multiple comparison test were performed to evaluate statistical significance. Values <0.05 (*), <0.01 (**), and <0.001 (***) were considered statistically significant.

## 3. Results

### 3.1. Differential Expression Levels of KRTs in Primary Human Hepatocytes

**Figure 1:**
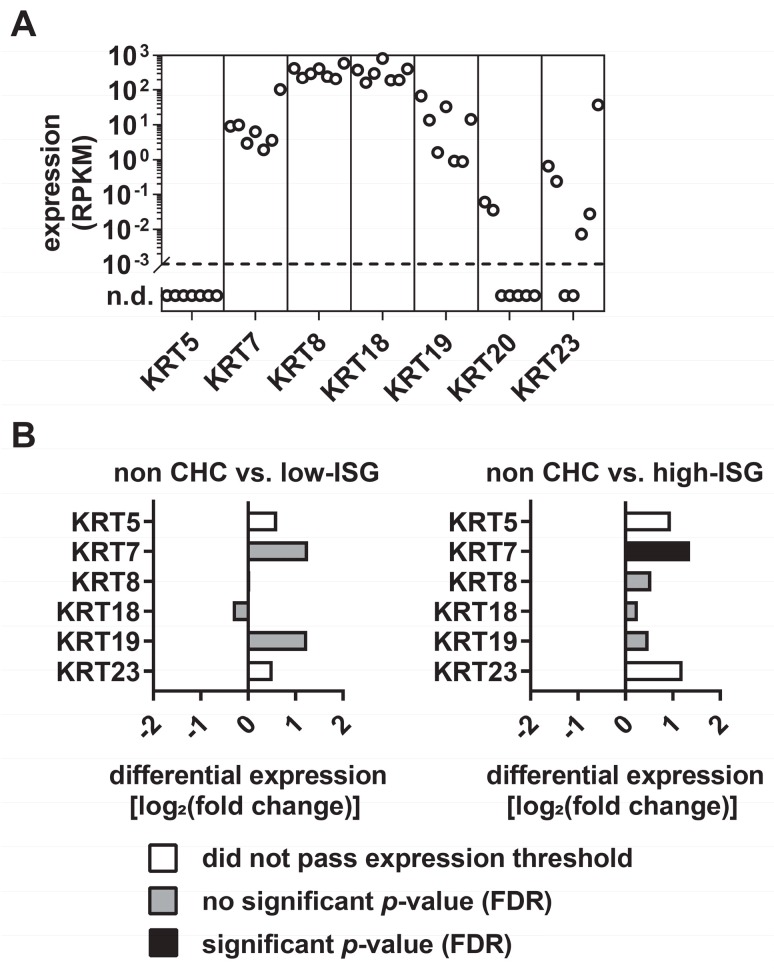
The expression of keratin 23 in primary human hepatocytes varies between different donors. (**A**) Gene expression analysis of keratins in primary human hepatocytes. Shown are the reads per kilobase million (RPKM) values of keratins from seven independent donors. (**B**) Differential expression of keratins in chronic hepatitis C patients (CHC) with low and high interferon-stimulated genes (ISG) induction compared with HCV-negative patients. Depicted are the differential expression of keratins in CHC patients with a low ISG response, compared with HCV-negative patients (left) and the differential expressions of keratins in CHC patients with a high ISG response compared with HCV negative patients (right). (FDR, false discovery rate).

First, we explored the expression levels of various KRTs in primary human hepatocytes (PHHs) from seven different donors (Tegtmeyer and Vieyres, manuscript in preparation: RNA-seq data set accession number GSE132548). As expected, transcriptomic analyses revealed the highest mRNA levels for the hepatocyte-specific keratins KRT8 and KRT18. In addition, KRT7 and KRT19, which are characteristic for hepatic progenitor cells [28], were expressed at a lower level, while KRT5 and KRT20 mRNA levels were under a threshold of 0.1 reads per kilobase million (RPKM) or undetectable (Figure 1A). Notably, the mRNA levels of KRT7, KRT8, KRT18, and KRT19 of the seven donors were correlated, whereas the KRT23 mRNA levels varied between different donors, with RPKM values ranging from 0–37 (Figure 1A). As KRTs are reported to be modulated in response to inflammation and disease, we aimed at exploring the expression of KRTs in a liver-pathology context. The observation that KRT8 and KRT18 variants associate with progression of fibrosis during CHC [29] encouraged us to use hepatitis C virus, a major cause of severe liver diseases, as a pathogenic model. Since CHC patients cluster into groups with a low or high intrinsic interferon response and this difference may be relevant for the molecular response in the human liver [30], we distinguished between low-interferon-stimulated genes (ISG) and high-ISG CHC patients. Subsequently, we compared the mRNA expression levels of KRTs of CHC patients with low or high ISG induction and HCV negative patients. In the livers of both low-ISG and high-ISG responders, the majority of the analyzed KRTs tended to be upregulated in CHC patients, compared with healthy donors (Figure 1B).

### 3.2. KRT23 Expression is Induced Ex Vivo in PHHs upon HCV Challenge 

**Figure 2:**
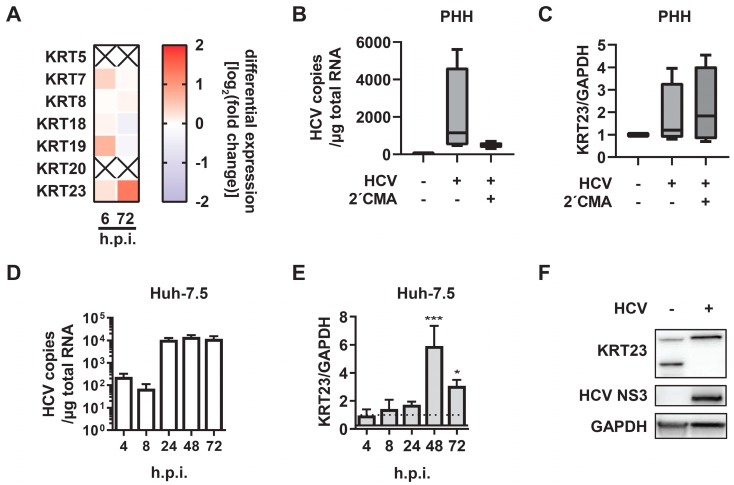
Keratin 23 is upregulated upon HCV challenge. (**A**) Heatmap of mRNA expression levels of keratins in primary human hepatocytes (PHH) upon HCV challenge (MOI 1). Shown are the mRNA expression levels of three independent donors 6 and 72 h post-infection. As a control for the differential gene expression, PHHs were treated with conditioned medium from Huh-7.5 cells and harvested at the same time points. (**B**) Comparison of HCV RNA copy numbers in primary human hepatocytes (PHHs) upon HCV infection (MOI 10) in the presence or absence of the polymerase inhibitor 2´CMA (10 μM). PHHs were infected with either mock or HCV Jc1 WT in the presence of dimethyl sulfoxide or 10 mM of 2′CMA and lysed 48 h post-infection. The HCV RNA copy numbers were determined by RT-qPCR and normalized to total RNA. (**C**) Comparison of KRT23 relative mRNA levels in mock-, HCV-infected (MOI 10), and 2′CMA-treated (10 μM) PHHs 48 hpi. KRT23 mRNA levels were normalized to GAPDH mRNA levels as determined by RT-qPCR. (**D**) Comparison of HCV RNA copy numbers in Huh-7.5 cells upon HCV infection (MOI 1). Huh-7.5 were left uninfected (mock) or infected with HCV Jc1 WT and lysed at the indicated time points. The HCV RNA copy numbers were determined by RT-qPCR and normalized to total RNA. (**E**) Analysis of KRT23 relative mRNA level induction in the time course of HCV infection (MOI 1). KRT23 mRNA levels were normalized to GAPDH mRNA levels as determined by RT-qPCR. The KRT23/GAPDH ratio of mock infected samples was arbitrarily set as one. (**F**) Western blot analysis of KRT23 protein levels in Huh-7.5 cells upon HCV challenge.

As we detected the regulation of KRTs in CHC patients, and taking into account that only the minority of hepatocytes are HCV infected during a chronic infection, we aimed to decipher whether KRTs are upregulated as a direct consequence of HCV infection. Therefore, PHHs were isolated from human liver specimens, infected with cell culture-derived HCV Jc1 WT, and subjected to transcriptomic analysis (Tegtmeyer and Vieyres, manuscript in preparation: RNA-seq data set accession number GSE132548). The expression of the majority of the analyzed KRTs was slightly affected, but intriguingly, we observed a specific induction of KRT23 expression 72 h post-infection (Figure 2A). Therefore, we aimed to analyze the KRT23 regulation upon HCV challenge in more detail and setup experiments with both primary cells and hepatoma cell-lines. We infected PHHs with a high MOI of cell culture-derived Jc1 virus and treated the cells with the HCV replication inhibitor 2’C-methyl-adenosine (2′CMA) as a control. Forty eight hours post-infection, the viral titer yielded 1 *×* 10^4^ TCID*_50_*/mL in Jc1-infected PHH, and HCV copy numbers and KRT23 mRNA expression levels were quantified by RT-qPCR (Figure 2B,C). In line with the transcriptomics data, KRT23 was upregulated in PHHs upon HCV challenge and in addition in 2′CMA-treated and HCV infected PHHs (Figure 2C). Thus, the data indicated that HCV challenge induced the expression of KRT23, even in the presence of 2′CMA. To evaluate the time course of KRT23 expression upon HCV challenge, we infected human hepatoma cells, which support the full HCV life-cycle. In these cells, we monitored the HCV RNA copy numbers and observed the highest HCV RNA levels from 24 until 72 h post-infection (Figure 2D). Importantly, KRT23 mRNA levels were most elevated at later time points (Figure 2E), indicating a correlation between HCV infection duration and KRT23 mRNA expression. Next, we investigated the modulation of KRT23 protein levels in human hepatoma cells upon HCV challenge. In the mock challenged cells, we detected two bands at ~33 kDa and ~48 kDa, representing the two KRT23 isoforms KRT23-001 (48 kDa) and KRT23-005 (33kDa) (Figure 2F). In contrast, we identified only one KRT23 band at ~48 kDa with higher intensity in the HCV challenged cells compared with the mock challenged cells (Figure 2F), indicating an HCV-induced alteration of the KRT23 protein levels. Taken together, the obtained data indicated that KRT23 was directly induced by HCV challenge in PHHs and hepatoma cells. 

### 3.3. KRT23 Plasma Levels Decrease Concomitant with HCV Clearance upon Anti-HCV Therapy

**Figure 3:**
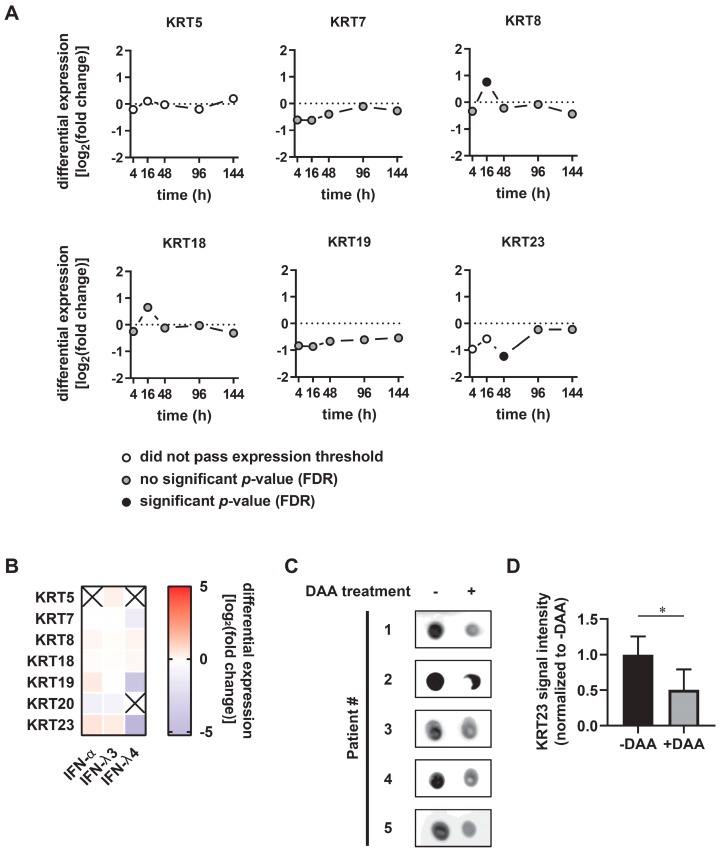
Keratin 23 serum levels decrease in response to direct-acting antiviral (DAA)-mediated viral clearance. (**A**) Keratin expression levels of pre-treatment and post-treatment liver biopsies of chronic hepatitis C patients at indicated time points after PEGylated interferon-α/ribavirin treatment. Raw data were obtained from Boldanova et al. [30]. (**B**) Heatmap of mRNA expression levels of keratins in primary human hepatocytes (PHHs) upon IFN-α (100 IU/mL), IFN-λ3 independent donors 6 h post-treatment. mRNA. (**C**) Keratin 23 sera levels of chronic hepatitis C patients (*n* = 5) pre- and post-DAA therapy. Patient sera, obtained prior to DAA treatment and post-viral clearance were assessed for the keratin 23 content via dot blot analysis. (**D**) Quantification of keratin 23 signals of the dot blot analysis (C). Keratin 23 level prior to DAA treatment was arbitrarily set as one. Shown are mean values ± standard deviation from five donors (unpaired two-tailed Student’s *t*-test; * *p* < 0.05).

pegIFN-α/ribavirin was the first choice of anti-HCV treatment for more than 25 years until it was replaced by direct acting antivirals (DAAs) in 2014 [13]. Whereas IFN induces a broadly antiviral state within the cell by the induction of 100s of interferon stimulated genes and thereby modulates the host innate immunity [31], DAAs exclusively target the viral proteins. We aimed to determine how these different therapy strategies affect the expression of KRTs, using publically-available datasets deposited in sequence databases and novel data generated from patient material. Previously, no modulation of KRTs resulting from the response to interferon treatment had been reported. Since KRT expression levels were increased in CHC patients, we asked whether IFN treatment modulates their expression in vivo. KRT5, KRT7, KRT18, and KRT19 mRNA levels were not affected during the first six days of pegIFN-α/ribavirin treatment (Figure 3A). In contrast, KRT8 was significantly upregulated 16 h post-treatment, whereas KRT23 mRNA significantly dropped 48 h post-treatment (Figure 3A). To evaluate the IFN-mediated regulation of KRT expression in the absence of a pathogen, PHHs were isolated from human liver specimen, treated with IFN-α in addition to IFN-λ3 and IFN-λ4 for 6 h, and subjected to transcriptomic analysis [15]. The modulation of KRTs was modest upon IFN-α and IFN-λ3, whereas IFN-λ4 treatment led to reduced mRNA levels of KRT19 and KRT23 (Figure 3B). Taken together, the obtained data indicate that pegIFN-α in vivo and IFN-α ex vivo are not key modulators of KRT23 expression. 

KRT23 is not only an intracellular protein, but also known to be released into the plasma. Previously, an elevation of KRT23 levels in the plasma of liver cirrhosis patient was observed when compared with healthy liver subjects [8]. Therefore, the question arose whether clearance of an HCV infection may lead to decreased levels of KRT23 in the plasma. Subsequently, we collected patient plasma of HCV-infected patients before the start of DAA treatment and after clearance of the infection and analyzed the KRT23 levels by immunoblotting. Notably, we observed significantly decreased levels of KRT23 concomitant with HCV RNA clearance upon DAA treatment (Figure 3C,D). These results strongly support the hypothesis that HCV directly induces the elevation of KRT23 in the plasma and furthermore indicate that the HCV-induced elevation of KRT23 in the plasma is reversible by DAA treatment.

### 3.4. Ectopically-Expressed KRT23 Facilitates the HCV Life Cycle Progression

**Figure 4:**
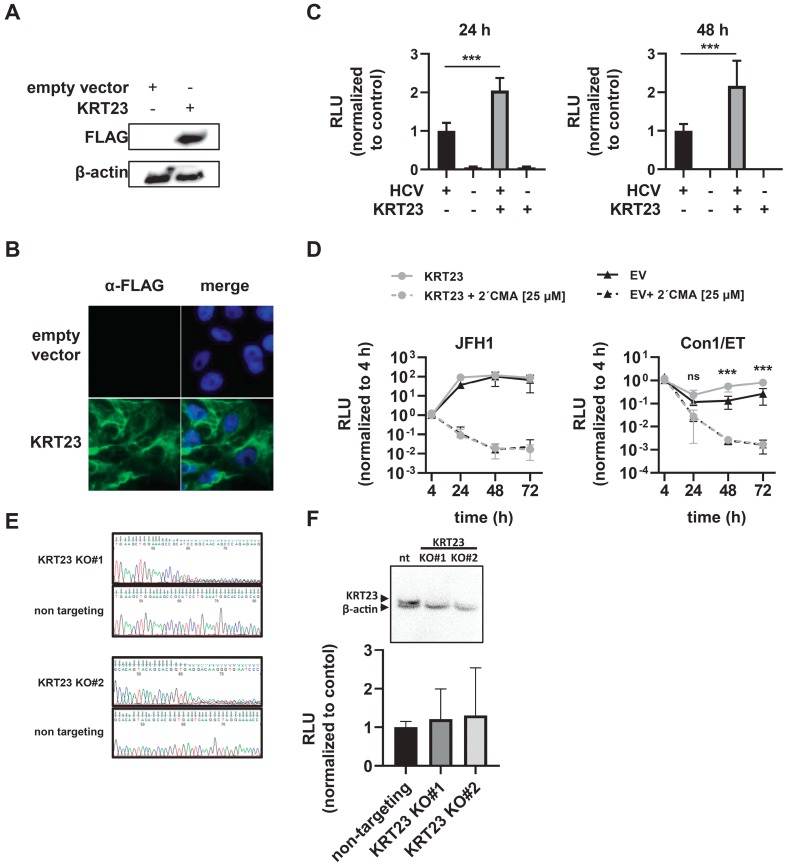
Expression of Keratin 23 facilitates HCV life cycle progression. (**A**) Western blot of Huh-7.5 cells stably expressing an empty vector (empty vector Huh-7.5) or 3xFLAG-KRT23 (KRT23 Huh-7.5). (**B**) Subcellular localization of KRT23 in KRT23 expressing Huh-7.5 cells. Cells were stained for 3xFLAG-tagged KRT23 (green), and nuclear DNA was stained with DAPI (blue). (**C**) Infection levels in empty vector and KRT23-expressing Huh-7.5 cells upon infection with HCV reporter virus JcR2a. Cells were infected with HCV JcR2a, and replication levels were determined by *Renilla* luciferase activity assay 24 h and 48 h post-infection. (**D**) HCV RNA replication efficiency of subgenomic replicons JFH1 NS3-3′and Con1/ET in empty vector and KRT23 Huh-7.5 cells. The firefly luciferase activity assay was performed to determine HCV RNA replication at the indicated time points. (C,D) Depicted are mean values ± standard deviation from three independent experiments (one-way ANOVA adjusted with Dunnett’s multiple comparison test; * *p* < 0.05; *** *p* < 0.001; ns: non-significant). (**E**) Editing of the target sequences in the designated KO cell lines. For validation, genomic DNA was sequenced to validate the loss of the consensus sequence in KO cell lines, compared with the non-targeting control. (**F**) Western blot of KRT23 KO Huh-7.5 cells and infection levels in non-targeting and KRT23 KO Huh-7.5 cells upon infection with HCV reporter virus JcR2a. Cells were infected with HCV JcR2a, and replication levels were determined by *Renilla* luciferase activity assay 48 h post-infection. (C,D,F) Depicted are mean values ± standard deviation from three independent experiments (one-way ANOVA adjusted with Dunnett’s multiple comparison test; * *p* < 0.05; *** *p* < 0.001; ns: non-significant).

To evaluate a potential pro- or anti-viral effect of KRT23, we generated Huh-7.5 cells expressing an empty vector or 3xFLAG-KRT23 (KRT23 Huh-7.5). First, we validated the expression of the KRT23 protein by immunoblotting and immunofluorescence staining (Figure 4A,B). Here, we observed filamentous structures for the ectopically-expressed KRT23 (Figure 4B). To see whether the KRT23 expression influences the HCV life cycle, we infected either empty vector or KRT Huh-7.5 cells with the HCV Jc1 *Renilla*-reporter virus. We observed a two-fold increased luciferase activity in cells ectopically expressing KRT23 at 24 h and 48 h post-infection, indicating a proviral effect of elevated KRT23 levels (Figure 4C). To analyze whether KRT23 expression influences the replication of HCV, we transfected subgenomic replicons of HCV JFH1 and Con1 into the empty vector and KRT23 Huh-7.5 cells. In line with the infection assay, the presence of KRT23 led to increased relative light units (RLU) counts (Figure 4D), supporting the hypothesis that KRT23 facilitates HCV replication.

To investigate the role of endogenous KRT23 in hepatoma cells, we generated two independent CRISPR/Cas9-mediated knockout (KO) cell lines of KRT23 with guide RNAs targeting the positive strand of exon 2 (KO #1) and the negative strand of exon 6 (KO #2). To validate the successful editing of the target sequences, we sequenced the genomic DNA, showing the loss of the consensus sequence, compared with the non-targeting control (Figure 4E). Furthermore, we proofed the KO by immunoblotting (Figure 4F). Infection experiments with the HCV Jc1 *Renilla*-reporter virus did not show an altered luciferase activity in the designated KO cell lines (Figure 4F), indicating that KO of KRT23 does not affect the viral life cycle in the tested hepatoma cell line. Taken together, KRT23 demonstrated a non-essential, but supportive HCV-induced host factor for the HCV life cycle.

## 4. Discussion

In the last few years, the classical role of KRTs in forming intermediate filaments was expanded by studies demonstrating that KRTs influence a variety of cellular processes including cell signaling, apoptosis, and stress responses [1,4,32,33]. Recently, the dramatic changes in KRT23 expression levels in the context of liver disease were reported [8,34]. In this study, we analyzed its role in the life cycle of the hepatotropic pathogen HCV and consequently discovered that KRT23 is an HCV-induced pro-viral factor.

In our initial experiments, in which we analyzed the expression of KRTs in PHHs, we observed the highest expression levels for the hepato-characteristic KRT8 and KRT18 and robust levels for hepatocyte progenitor characteristic KRT7 and KRT19 (Figure 1A). Since the expression of distinct KRTs is restricted to specific tissues and the substitution of primary KRTs by others occurs in a tissue-specific manner, we observed an extreme variable expression of KRT23 in hepatocytes (Figure 1A). Further analysis of KRT expression in a CHC patient cohort revealed an upregulation of KRTs, including KRT23, in CHC patients compared with uninfected patients (Figure 1B). This upregulation might indicate a stress-induced upregulation of KRTs and/or a further substitution of the primary KRTs by other ones in a disease-dependent manner. However, transcriptomics data from liver biopsies do not take into account that the majority of the cells in the sample are HCV negative, and rather indicate a global alteration caused by HCV infection. Furthermore, Guldiken and colleagues observed that KRT23 upregulation is independent of disease etiology, but dependent on disease progression [8]. However, subsequent analysis of PHHs and hepatoma cells supported the hypothesis that KRT23 is upregulated in an HCV-dependent manner on a single cell level (Figure 2C,E). Notably, elevated KRT23 mRNA were also detected in 2´CMA-treated and HCV-infected cells (Figure 2C), pointing to a potential replication-independent induction of KRT23. By analyzing the KRT23 protein levels in hepatoma cells, we detected the disappearance of the designated 33-kDa KRT23 isoform, but increased signal intensities of the 48 kDa KRT23 isoform in the HCV challenged cells compared with the mock challenged cells. These results suggest an HCV-mediated shift of KRT23 isoform abundance and a preferential expression of the 48-kDa KRT23 isoform. KRT23 is reported to be induced in a peroxisome proliferator-activated receptor alpha (PPARα)-dependent and MYC amplified fashion, highlighted by several PPARα and MYC binding sites, as well a PPARα-deficient mouse model [35]. Interestingly, several studies reported an HCV-core induced activation of and interference with PPARα [36,37,38], as well as MYC [39], which subsequently might explain the induction of KRT23 upon HCV challenge. 

Following experiments and analysis of publically-available transcriptomics data revealed that expression levels of most KRTs are not modulated by pegIFN-α therapy (Figure 3A). In contrast, we detected decreased KRT23 expression 48 h post-IFN injection (Figure 3A), which is associated with the early phase of antiviral efficiency of IFN treatment [40], indicating a correlation between KRT23 mRNA level decrease and the drop of the viral load. Importantly, IFN-α treatment of PHHs in the absence of HCV did not alter the KRT23 mRNA levels (Figure 3B), indicating that IFN-α has no pronounced direct effect on KRT23 expression. By analyzing the KRT23 levels of HCV-positive patients, we observed a decrease of KRT23 sera levels caused by DAA treatment concomitant with viral clearance (Figure 3C). Taking the findings of Guldiken and colleagues that KRT23 is a stress-induced marker into account, the decline of KRT23 levels highlights the regenerative potential of the liver and the potential of DAA to revert the progression of liver diseases. 

Experimental settings with both viral particles and subgenomic replicons of HCV indicated that forced expression of KRT23 positively affects the replication of HCV (Figure 4C,D). The non-affected replication capacity in KRT23 KO cells may be based on phenotypic compensation by counter-regulation of other KRTs or could also indicate that KRT23 is not essential for the HCV life cycle progression. Regarding a potential interplay between HCV and KRT23, Liffers and colleagues observed direct interaction of KRT23 with KRT8, KRT18, plectin1, 14-3-3ε, heat shock protein (HSP) 60, and HSP70 [41]. Intriguingly, the three latter proteins are reported to directly interfere with HCV proteins [42,43,44]. For example, Gonzales and colleagues reported that HSP70 interacts with NS5A and treatment with Quercetin, an HSP expression inhibitor, led to decreased virus production [45], pointing to a role of HSPs in the HCV life cycle. 14-3-3ε, which is known to interfere with several cell signaling components, does not only interact with KRT23, but was also shown to interact with the HCV entry receptor CD81 and HCV core [46,47]. Taken together, the interference of KRT23 interactors with the HCV life cycle supports our hypothesis of a potential pro-viral role for KRT23.

In summary, our data indicate that KRT23 is an HCV-induced pro-viral host factor. Further studies will help to investigate the HCV-mediated regulation of KRT23, giving insight into the molecular mechanisms of how KRT23 supports the HCV life cycle. Of note, the decrease in KRT23 plasma levels concomitant with HCV RNA clearance highlights the regenerative potential of the liver after HCV infection. Thus, the idea to use KRT23 as a serum marker to monitor hepatic disease progression may be extended by the usage of KRT23 to monitor liver regeneration.

## Appendix A

**Table A1:** Sequences of the oligonucleotides used for the single-guide RNA (sgRNA) vector cloning and for the sequencing of the genomic DNA (gDNA) of the CRISPR/Cas 9 KO cell lines.

**KRT23 KO Oligos**	
**specification**	**sequence**
KRT23_Oligo #1_fwd	GTCGTCTCCCACCGGCTGGTGCCATTTCAGGATG GTTTCGAGACGTG
KRT23_Oligo #1_rev	CACGTCTCGAAACCATCCTGAAATGGCACCAGCCGGTGGGAGACGAC
KRT23_Oligo #2_fwd	GTCGTCTCCCACCGCAGTACAGCACGGTGAGTCAGTTTCGAGACGTG
KRT23_Oligo #2_rev	CACGTCTCGAAACTGACTCACCGTGCTGTACTGCGGTGGGAGACGAC
**KRT23 KO gDNA primers for sequencing**	
**specification**	**sequence**
KRT23_gRNA1_left	ACCATGCAGAATCTCAACGAC
KRT23_gRNA1_right	TGGAAATTGTCTTCTGGACTCA
KRT23_gRNA2_left	ACTGTGCAGAGCAGACAAGGT
KRT23_gRNA2_right	GGAAAGTGAGAGTTGTCCCAAG
